# Clinical determinants of agreement and discordance between stress SPECT and invasive coronary angiography

**DOI:** 10.3389/fcvm.2026.1701610

**Published:** 2026-02-05

**Authors:** Matan Danon, Nitzan Shabat Cohen, Saar Ashri, Yehuda Warszawer, Yonathan Hasin

**Affiliations:** 1 Adelson School of Medicine, Ariel University, Ariel, Israel; 2 School of Public Health, Gray Faculty of Health & Medical Sciences, Tel Aviv University, Tel Aviv, Israel; 3 Departments of Urology & Pediatric Urology, Shaare Zedek Medical Center, Jerusalem, Israel; 4 Meuhedet Health Maintenance Organization, Tel Aviv, Israel

**Keywords:** clinical model, coronary artery disease, diagnostic agreement, invasive coronary angiography, SPECT

## Abstract

**Background:**

Stress single-photon emission computed tomography (SPECT) myocardial perfusion imaging offers a non-invasive alternative for invasive coronary angiography (ICA) in diagnosing coronary artery disease (CAD), however often yields inconclusive results. This study aimed to characterize patterns of agreement and discordance between stress SPECT and ICA, and to evaluate potential risk stratification.

**Methods:**

We retrospectively analyzed 915 patients with suspected CAD who underwent stress SPECT followed by ICA within three months (enrollment: 2019–2020). Clinical, demographic, and imaging data were extracted from medical records. Variables associated with diagnostic agreement were identified using univariate logistic regression. A predictive model combining SPECT results with clinical variables was developed using backward stepwise logistic regression and evaluated by AUC-ROC.

**Results:**

Diagnostic agreement between stress SPECT and ICA was observed in 624 patients (68.2%), while 291 patients (31.8%) demonstrated discordant results. Agreement was associated with use of nitrates (OR 3.18, 95% CI 1.31–7.73), antiplatelet therapy (OR 2.57, 95% CI 1.86–3.56), renal failure (OR 2.34, 95% CI 1.43–3.84), and type II diabetes mellitus (OR 1.78, 95% CI 1.28–2.48), whereas female sex (OR 0.50, 95% CI 0.34–0.73), smoking (OR 0.72, 95% CI 0.50–1.03), and higher body mass index (BMI; OR 0.95 per kg/m², 95% CI 0.92–0.99) were associated with disagreement. The final multivariable model included stress SPECT results, sex, BMI, smoking status, serum creatinine, renal failure, type II diabetes mellitus, and use of antiplatelets and nitrates and demonstrated improved discrimination compared with SPECT alone (AUC 0.72 vs. 0.54, *p* < 0.001).

**Conclusion:**

In this exploratory study, clinical factors were associated with an agreement between stress SPECT and ICA. Incorporating clinical context alongside SPECT findings may help inform risk stratification.

## Introduction

Coronary artery disease (CAD) is a leading cause of mortality worldwide and requires timely and accurate diagnosis and treatment (1, 2). While invasive coronary angiography (ICA) remains the gold standard for diagnosing CAD, its invasive nature entails procedural risks and resource-intensive requirements, necessitating judicious utilization (3). Indeed, prior studies have shown that up to 40% of elective ICAs reveal no significant obstructive coronary disease, highlighting concerns about overuse and the limited diagnostic yield in real-world settings (2).

Several noninvasive imaging modalities, such as stress single photon emission computed tomography (SPECT) myocardial perfusion imaging (MPI), are used to assess inducible myocardial ischemia. By detecting regional disparities in myocardial blood flow during stress conditions SPECT enables the identification of myocardial ischemia (4). Although SPECT and other noninvasive tests are being used for diagnosing CAD (5), SPECT showed limited accuracy in real world settings (67%–69%) (6). Moreover, stress SPECT results often diverge from ICA findings, resulting in discordant findings that pose challenges to clinical interpretation (7, 8). This mismatch reflects the fundamental difference between the two modalities: SPECT assesses myocardial perfusion under stress, capturing functional impairment, whereas ICA provides anatomical visualization of coronary artery stenosis. These distinct approaches may lead to disagreement, particularly in cases of microvascular dysfunction or balanced ischemia.

While ICA remains a necessary tool in CAD diagnosis, it is essential to recognize the strengths of noninvasive imaging methods such as SPECT. Compared to other modalities like coronary computed tomography angiography (CCTA) or cardiac MRI, SPECT is generally more accessible and widely available in routine clinical settings (9). It requires less specialized infrastructure, is performed in a larger number of medical centers, and does not rely on iodinated contrast or advanced breath-holding protocol, factors that may complicate CCTA or MRI use. As such, SPECT is often more easily integrated into routine diagnostic workflows. This is reflected in current clinical guidelines, which recommend non-invasive imaging—commonly SPECT—for patients with intermediate pre-test probability of CAD (2, 10). These recommendations emphasize SPECT's central role in clinical practice and underscore the importance of optimizing its diagnostic performance.

Within guideline-optimized care, patients may still undergo ICA without prior noninvasive testing (11), or even after a normal SPECT result (12). While such decisions align with current clinical protocols, they may contribute to potentially unnecessary procedures or delays in definitive management in selected cases. The diagnostic inefficiencies, including false positives and negatives, increase patients' exposure to procedural risks and complications (13). In this study, we explore the relationship between routinely-used clinical factors and agreement or disagreement between the two modalities.

## Methods

This retrospective cohort study comprised consecutive patients of “Meuhedet”, the third-largest health maintenance organization (HMO) in Israel, who underwent stress SPECT, followed by ICA within a 3-month period between January 1, 2019, and December 31, 2020, at 29 certified imaging centers across Israel. Indications for ICA in this cohort were primarily based on the attending physician's clinical judgment, which integrated the patient's cardiovascular risk profile as well as clinical presentation. Individual-level patients' data were obtained from the electronic medical records, encompassing personal, demographic, clinical information, and results from both invasive and non-invasive diagnostic tests. This study was approved by the institutional review board.

### Imaging procedures

Stress SPECT imaging (via treadmill exercise and/or pharmacological agents—dipyridamole/papaverine) was performed by specialized technicians and analyzed by specialized cardiologists. Tests were considered positive when showing any reversible perfusion defect. Additionally, we defined angiographically obstructive CAD as a visually estimated diameter stenosis ≥60% on ICA. Because quantitative coronary angiography (QCA) and coronary physiology (i.e., functional flow reserve) were unavailable, this threshold was selected *a priori* to improve specificity in the setting of known systematic overestimation of stenosis severity by visual assessment vs. QCA (14). All reported results of individual cases were reviewed retrospectively to determine the agreement or disagreement between the SPECT or ICA results. The patient data were categorized into two distinct groups: “Agreement,” encompassing cases with concordant results (positive SPECT/positive ICA or negative SPECT/negative ICA), and “Disagreement” (positive SPECT/negative ICA or negative SPECT/positive ICA).

### Data collection

Personal and demographic data included age (years), sex, and body mass index (BMI, kg/m^2^). For descriptive analyses, BMI was categorized into four groups: 14–18.49, 18.5–24.9, 25–29.9, and 30–45; BMI was modelled as a continuous variable. Behavioral characteristics included active smoking status. Clinical diagnoses comprised of a broad range of cardiovascular and non-cardiovascular conditions, including history of acute coronary syndrome (ACS), angina pectoris, atrial fibrillation, congestive heart failure (CHF), peripheral vascular disease (PVD), chronic obstructive pulmonary disease (COPD), type 2 diabetes mellitus (DM2), hypertension, dyslipidemia, renal failure, anemia, iron deficiency anemia, liver dysfunction, and a family history of premature cardiovascular disease. All diagnoses were extracted from ICD-9 coded electronic medical records. Renal failure was defined by either a diagnosis code or by an estimated glomerular filtration rate (eGFR) <60 mL/min/1.73 m^2^. Iron deficiency anemia was ascertained based on either a diagnosis code or evidence of iron supplementation. Dyslipidemia was defined according to diagnosis codes or laboratory criteria based on the 2018 cholesterol management guidelines (15). Laboratory variables included serum creatinine (mg/dL), hemoglobin (g/dL), platelet count (×10^3^/μL), and hemoglobin A1c (HbA1c, %). Medication data were obtained from prescription records and grouped by therapeutic indication: anti-hyperlipidemic agents (including statins, bezafibrate, and ezetimibe), antihypertensive medications (including angiotensin-converting enzyme inhibitors, beta-blockers, alpha antagonists, calcium channel blockers, and angiotensin receptor blockers), diuretics (including thiazides, loop diuretics, and mineralocorticoid receptor antagonists), antidiabetic medications (including sulfonylureas, metformin, glucagon-like peptide-1 analogues, and sodium-glucose cotransporter-2 inhibitors), as well as antiplatelets, anticoagulants, nitrates, non-steroidal anti-inflammatory drugs, and psychiatric medications, including selective serotonin reuptake inhibitors and serotonin–norepinephrine reuptake inhibitors.

### Statistical analysis

Statistical analyses were conducted using IBM SPSS Statistics, version 26 (IBM Corp., Armonk, N.Y., USA), and MedCalc for diagnostic test performance metrics. Descriptive statistics included mean and standard deviation (SD) for continuous variables and frequency (%) for categorical variables, and were stratified according to agreement group. Univariate logistic regression models were constructed to examine the associations between clinical characteristics and modalities agreement. Associations were expressed as odds ratios (OR) with 95% confidence intervals (CI); *p*-values were derived from the Wald Chi-square test. We subsequently constructed multivariable adjusted logistic regression models with and without backward stepwise selection with positive ICA as the outcome. The initial model included SPECT results alone to predict ICA findings (Model 1), followed by inclusion of potential confounding demographic (Model 2) and clinical factors (Model 3). We further constructed a backwards-stepwise logistic regression model that removed any variable that did not contribute to predictive performance, based on the Akaike information criterion (16) (Model 4). Sensitivity, Specificity, Accuracy, positive predictive value (PPV), and negative predictive value (NPV) were calculated for each model. The discriminatory ability of the models was compared through receiver operating characteristic (ROC) curve analysis, with the area under the curve (AUC) serving as a measure of the model's overall accuracy. To formally assess differences in AUC between models, pairwise comparisons were performed using the DeLong test for correlated ROC curves (17). Due to missing information, we excluded HbA1c from the main analysis and conducted a separate sensitivity analysis of Models 3 + 4 with HbA1c included.

## Results

We analyzed data of 915 patients, mean age 65.6 (SD 10.2), 192 (20.98%) were women. Of them, 574 (62.7%) had both positive SPECT and ICA, 50 (5.5%) had negative SPECT and ICA, 230 (25.1%) had positive SPECT and negative ICA, and 61 (6. 7%) had negative SPECT and positive ICA (Supplementary Table S1). A comprehensive description of baseline demographic, clinical, and treatment characteristics across these four diagnostic subgroups is presented in Supplementary Table S2. The agreement group comprised of a total of 624 (68.2%) patients, while 291 (31.8%) were in the disagreement group. At baseline, patients in the agreement group were more likely to be male and had higher prevalence of ACS, renal failure, and hypertension. They were also more frequently treated with antiplatelet and anti-hyperlipidemic medications (Table 1).

**Table 1:** Baseline characteristics of study participants by agreement groups.

Variable	SPECT & ICA agreement^a^	SPECT & ICA disagreement^a^	Sig.
Demographic characteristics			
Age (year)	66.02 (9.70)	64.65 (11.06)	0.06
Female sex	115 (18.4%)	77 (26.5%)	0.01
Physical characteristics			
BMI (kg/m^2^)			
14–18.49	2 (0.3%)	0 (0%)	
18.5–24.9	80 (13.2%)	40 (14.3%)	
25–29.9	258 (42.4%)	111 (39.6%)	
30–45	268 (44.1%)	129 (46.1%)	
History of acute coronary syndrome	87 (13.9%)	26 (8.9%)	0.03
Angina Pectoris	492 (78.8%)	237 (81.4%)	0.36
Atrial Fibrillation	62 (9.9%)	43 (14.8%)	0.03
Congestive Heart Failure	36 (5.8%)	19 (6.5%)	0.65
COPD	93 (14.9%)	47 (16.2%)	0.63
Diabetes mellitus Type 2	312 (50%)	124 (42.6%)	0.04
Familial history of cardiovascular disease	67 (10.7%)	38 (13.1%)	0.31
Hypertension	497 (79.6%)	216 (74.2%)	0.07
Smoking	153 (24.5%)	78 (26.8%)	0.46
PVD	102 (16.3%)	36 (12.4%)	0.12
Anemia	203 (32.5%)	90 (30.9%)	0.63
Liver Dysfunction	174 (27.9%)	88 (30.2%)	0.46
Renal failure	152 (24.4%)	45 (15.5%)	0.00
Iron deficiency anemia	84 (13.5%)	34 (11.7%)	0.46
Dyslipidemia	438 (70.2%)	193 (66.3%)	0.24
Laboratory findings			
Creatinine Serum (mg/dL)	1.11 (0.79)	1.07 (0.94)	0.50
Hemoglobin (g/dL)	14.00 (1.69)	14.11 (1.72)	0.35
Platelets (×10³/μL)	233.08 (70.51)	233.43 (68.07)	0.95
Chronic medications			
NSAIDs	118 (18.9%)	52 (17.9%)	0.71
Antiplatelets	445 (71.3%)	172 (59.1%)	0.00
Anticoagulant	41 (6.6%)	26 (8.9%)	0.20
Nitrates	53 (8.5%)	15 (5.2%)	0.07
Anti-hyperlipidemic (combined)	481 (77.1%)	200 (68.7%)	0.01
Psychiatric Medication (combined)	76 (12.2%)	45 (38.5%)	0.17
Anti-diabetic (combined)	101 (16.2%)	33 (11.3%)	0.05
Anti-hypertensive (combined)	498 (79.8%)	222 (76.3%)	0.23
Diuretics (combined)	110 (17.6%)	53 (18.2%)	0.83

SPECT, single photon emission computed tomography; ICA, invasive coronary angiography; Sig., statistical significance; PVD, peripheral vascular disease; BMI, body mass index; COPD, chronic obstructive pulmonary disease; NSIADS, non-steroidal anti-inflammatory drugs.

a Data are mean (SD) or *n* (%).

Univariate associations (OR, 95% CI) between baseline clinical variables and agreement between stress SPECT and ICA showed that increasing age (OR 1.01, 95% CI 1.00–1.03), history of ACS (OR 1.65, 95% CI 1.04–2.62), DM2 (OR 1.35, 95% CI 1.02–1.78), use of antiplatelet therapy (OR 1.72, 95% CI 1.29–2.30), antihyperlipidemic treatment (OR 1.53, 95% CI 1.12–2.09), renal failure (OR 1.76, 95% CI 1.22–2.54), and higher HbA1c levels (OR 1.21 per % change, 95% CI 1.07–1.37) were associated with increased likelihood of diagnostic agreement. In contrast, female sex (OR 0.63, 95% CI 0.45–0.87) and atrial fibrillation (OR 0.64, 95% CI 0.42–0.97) were associated with disagreement between the modalities. Marginal associations were observed for hypertension (OR 1.36, 95% CI 0.98–1.89), chronic nitrate use (OR 1.71, 95% CI 0.95–3.08), and anti-diabetic treatment (OR 1.51, 95% CI 0.99–2.30), followed by body mass index (OR 0.97, 95% CI 0.94–1.00) and liver dysfunction (OR 0.89, 95% CI 0.66–1.21) (Table 2).

**Table 2:** Associations between clinical characteristics at baseline and an agreement between stress single photon emission computed tomography myocardial perfusion scan and invasive coronary angiography.

Variable	OR (95% CI)	*P* ^a^
Demographic characteristics		
Age (Year)	1.01 (1.00, 1.03)	0.06
Female sex	0.63 (0.45, 0.87)	0.01
Physical characteristics		
BMI (kg/m²)	0.97 (0.94, 1.00)	0.09
Acute Coronary Syndrome	1.65 (1.04, 2.62)	0.03
Angina Pectoris	0.85 (0.60, 1.21)	0.36
Atrial Fibrillation	0.64 (0.42, 0.97)	0.03
Chronic Heart Failure	0.88 (0.49, 1.56)	0.65
COPD	0.91 (0.62, 1.33)	0.63
Diabetes Mellitus Type 2	1.35 (1.02, 1.78)	0.04
Familial History of cardiovascular disease	0.80 (0.52, 1.23)	0.31
Hypertension	1.36 (0.98, 1.89)	0.07
Smoking	0.89 (0.65, 1.22)	0.46
PVD	1.38 (0.92, 2.08)	0.12
Anemia	1.08 (0.80, 1.45)	0.63
Liver Dysfunction	0.89 (0.66, 1.21)	0.06
Renal failure	1.76 (1.22, 2.54)	0.00
Iron deficiency anemia	1.18 (0.77, 1.80)	0.46
Dyslipidemia	1.20 (0.89, 1.61)	0.24
Laboratory findings		
Creatinine Serum (mg/dL)	1.07 (0.88, 1.29)	0.50
HbA1c (%)	1.21 (1.07, 1.37)	0.00
Hemoglobin (g/dL)	0.96 (0.88, 1.05)	0.35
Platelets (×10³/μL)	1.00 (1.00, 1.00)	0.95
Regular treatment		
NSAIDs	1.07 (0.75, 1.54)	0.71
Antiplatelets	1.72 (1.29, 2.30)	0.00
Anticoagulant	0.72 (0.43, 1.20)	0.20
Nitrates	1.71 (0.95, 3.08)	0.08
Anti-hyperlipidemic treatment	1.53 (1.12, 2.09)	0.01
Psychiatric Medications	0.76 (0.51, 1.13)	0.17
Anti-diabetic treatment	1.51 (0.99, 2.30)	0.05
Anti-hypertensive treatment	1.23 (0.88, 1.72)	0.23
Diuretics treatment	0.96 (0.67, 1.38)	0.83

SPECT, single photon emission computed tomography; ICA, invasive coronary angiography; Sig., statistical significance; PVD, peripheral vascular disease; BMI, body mass index; COPD, chronic obstructive pulmonary disease; NSIADS, non-steroidal anti-inflammatory drugs.

a *P* values are derived from Chi-square test.

**Figure 1:**
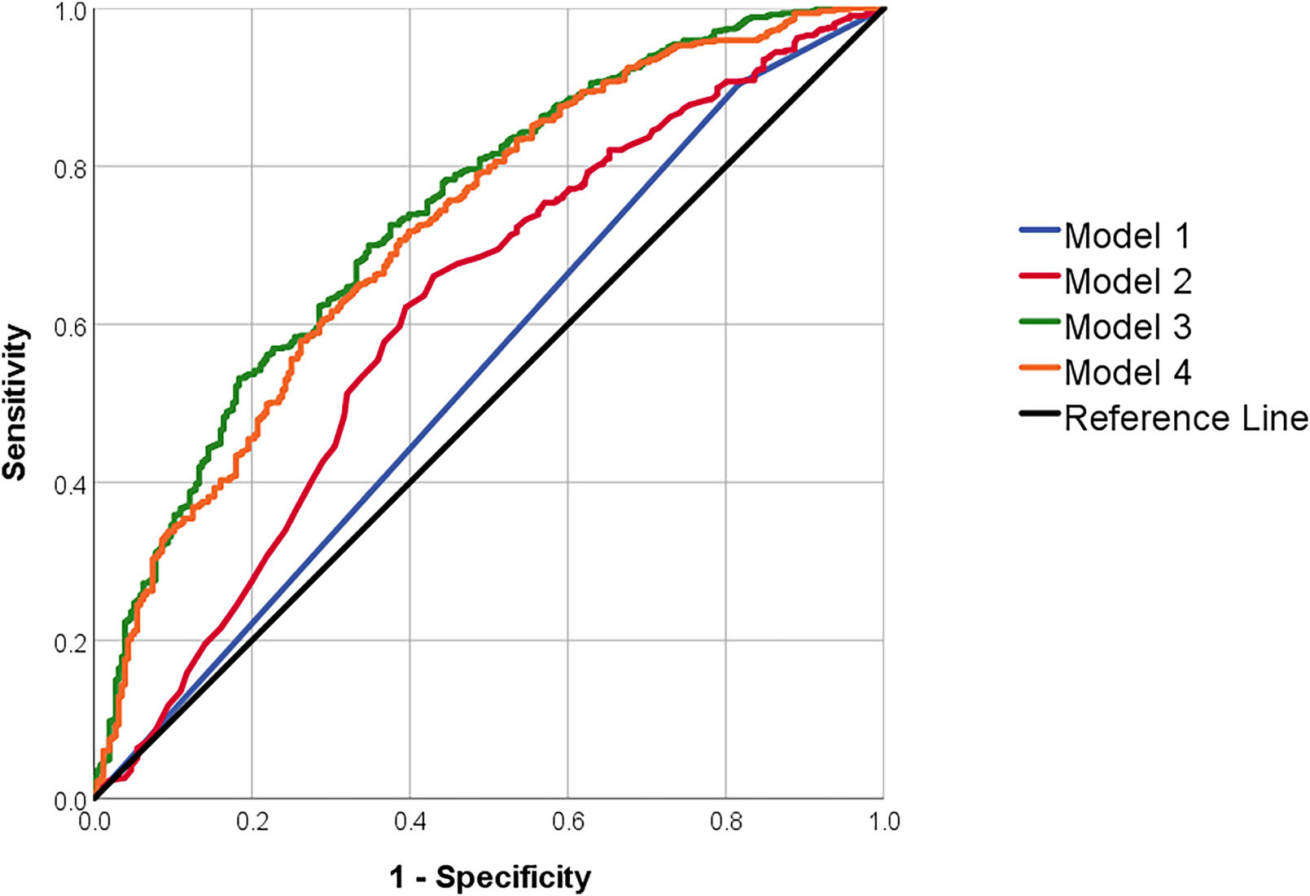
Receiver operating characteristic curves of models predicting positive invasive coronary angiography. Model 1 includes SPECT results alone; Model 2 adds demographic variables (age and sex); Model 3 incorporates all clinical variables; and Model 4 is a simplified model derived via backward stepwise logistic regression.

We evaluated the predictive performance of stress SPECT alone and in combination with clinical characteristics, in a total of four models, using ROC analysis (Table 3, Figure 1). Model 1, which included SPECT alone, yielded an AUC of 0.54 and an accuracy of 68.20%. Adding demographic factors (age and sex) in Model 2 improved the AUC and accuracy to 0.62 and 69.62%, respectively (*p* < .001). Model 3, which incorporated all clinical variables, further increased AUC to 0.74 and accuracy to 74.68% (*p* < .001). Model 4, constructed via backward stepwise logistic regression, achieved an AUC of 0.72 and an accuracy of 74.39% (Table 3, *p* < .001 vs. model 1). Sensitivity analysis that included HbA1c in Models 4 among individuals with available HbA1c measurement yielded comparable associations and predictive performance (Supplementary Table S3).

**Table 3:** Prediction of positive invasive coronary angiography results using stress single photon emission computed tomography myocardial perfusion scan and clinical data.

Variable^a^	SPECT only—Model 1	SPECT + personal characteristics—Model 2	SPECT + all clinical data—Model 3	SPECT + stepwise regression—Model 4
# of included subjects	915	915	869	871
single photon emission computed tomography	2.05 (1.37, 3.06)	1.98 (1.31, 2.99)	2.28 (1.42, 3.64)	2.34 (1.48, 3.70)
Demographic characteristics				
Age (years)		1.02 (1.00, 1.03)	1.00 (0.99, 1.02)	
Female gender		0.48 (0.35, 0.68)	0.40 (0.25, 0.62)	0.50 (0.34, 0.73)
Physical characteristics				
BMI cont. (kg/m²)			0.95 (0.92, 0.99)	0.95 (0.92, 0.99)
History of Acute Coronary Syndrome			1.17 (0.68, 2.01)	
Angina Pectoris			0.84 (0.56, 1.25)	
Atrial Fibrillation			0.61 (0.29, 1.30)	
Congestive Heart Failure			0.71 (0.35, 1.46)	
COPD			1.09 (0.68, 1.76)	
Diabetes mellitus Type 2			1.59 (1.10, 2.30)	1.78 (1.28, 2.48)
Familial history of cardiovascular disease			0.76 (0.46, 1.24)	
Hypertension			1.12 (0.71, 1.75)	
Smoking			0.63 (0.42, 0.94)	0.72 (0.50, 1.03)
PVD			1.57 (0.93, 2.66)	
Anemia			0.95 (0.62, 1.46)	
Liver Dysfunction			0.78 (0.54, 1.11)	
Renal failure			2.21 (1.29, 3.79)	2.34 (1.43, 3.84)
Iron deficiency anemia			0.83 (0.47, 1.47)	
Dyslipidemia			1.41 (0.97, 2.04)	
Laboratory findings				
Creatinine Serum (mg/dL)			0.76 (0.61, 0.95)	0.77 (0.62, 0.95)
Hemoglobin (g/dL)			0.92 (0.81, 1.05)	
Platelets (×10^3^/μL)			1.00 (1.00, 1.00)	
Regular treatment				
NSAIDs			1.35 (0.87, 2.09)	
Antiplatelets			2.20 (1.47, 3.28)	2.57 (1.86, 3.56)
Anticoagulant			1.12 (0.44, 2.82)	
Nitrates			3.20 (1.28, 8.01)	3.18 (1.31, 7.73)
Anti-hyperlipidemic treatment			1.39 (0.92, 2.10)	
Psychiatric Medication			0.82 (0.51, 1.32)	
Anti-diabetic treatment			1.24 (0.72, 2.14)	
Anti-hypertensive treatment			1.13 (0.70, 1.82)	
Diuretics treatment			0.74 (0.45, 1.22)	
Sensitivity (%)	90.39	97.64	92.33	93.32
Specificity (%)	17.86	6.07	32.42	29.18
Positive predictive value (%)	71.39	70.22	76.59	75.89
Negative predictive value (%)	45.04	53.12	63.85	64.66
Accuracy (%)	68.20	69.62	74.68	74.39
AUC	0.54	0.62	0.74	0.72

SPECT, single photon emission computed tomography; ICA, invasive coronary angiography; Sig., statistical significance; PVD, peripheral vascular disease; BMI, body mass index; COPD, chronic obstructive pulmonary disease; NSIADS, non-steroidal anti-inflammatory drugs.

a Associations between variables and positive ICA are presented in OR (95% CI) and are adjusted to all other variables included in the model.

## Discussion

In this study, we found that the likelihood for agreement between stress SPECT and ICA is associated with several demographic, behavioral and clinical factors. Moreover, models with clinical factors alongside stress SPECT result had better predictive performance compared with SPECT alone. A stepwise regression model showed that only a handful of factors are needed to reach better discrimination.

Our results with SPECT's accuracy of 68% in the diagnosis of significant epicardial CAD as shown by ICA corroborate with other real-world studies showing a similar accuracy of 67%–70% (6, 18). Further to the above, our analysis indicates that incorporation of clinical data may partially overcome the limitation of stress SPECT. The gap between SPECT and ICA results likely reflects the fundamental difference between functional and anatomical assessment: SPECT evaluates myocardial perfusion under stress, whereas ICA visualizes epicardial coronary anatomy. As a result, impaired perfusion detected by SPECT may arise from mechanisms not fully captured by angiography, including microvascular dysfunction or diffuse ischemia, which have been increasingly recognized in patients with ischemia in the absence of obstructive coronary artery disease (19). Emerging evidence further suggests that vascular inflammation, assessed by novel biomarkers such as the perivascular fat attenuation index (pFAI), may contribute to this discordance by impairing myocardial perfusion independently of epicardial stenosis severity (20, 21).

Several clinical characteristics may contribute to the reduced agreement between the modalities in the diagnosis of CAD. Indeed, female sex, obesity and smoking were significantly associated with a disagreement. SPECT MPI has been reported to have limited accuracy in women due to breast tissue and a smaller heart size (22) yet a meta-analysis of prospective studies did not reveal a significant difference in the diagnostic accuracy of SPECT MPI between women and men (23). Obesity, especially extreme, has been found to interfere with the diagnostic accuracy of SPECT due to Compton diffusion and attenuation artifacts (24). Furthermore, the diagnostic accuracy of non-attenuated SPECT, even when corrected (mainly for inferior wall), is lower in patients with BMI higher than 30 (25). Similarly, we found that smoking was associated with disagreement between the modalities. Smoking may potentially cause vasospastic angina and microvascular dysfunction, resulting in ischemia despite non-obstructive angiography (26). On the other hand, smoking may result in diffuse epicardial atherosclerosis, yielding false negative SPECT test (27). The main mechanisms underlined for smoking-induced vascular endothelial cell damage are oxidative stress, inflammation, and impaired Na^+^/K^+^ ATPase function (28).

Additionally, DM2, renal failure and treatment with antiplatelets and nitrates were associated with an agreement between SPECT and ICA. Indeed, DM2 is a known CAD risk factor (29), and the incidence of cardiovascular events and death are significantly higher in diabetic patients with MPI SPECT abnormalities (30). Chronic renal failure and elevated serum creatinine is also associated with a high prevalence of obstructive CAD (31), in line with our findings. Indeed, there is evidence on the value of SPECT MPI in the prediction of cardiovascular events in patients with CKD (32). Cardiovascular risk of patients with CKD is attributable to risk factors and comorbidities shared between CKD and CAD, including hypertension, dyslipidemia, and diabetes. Aside from shared risk factors (33), other, less-well-defined kidney-specific risk modifiers—such as disturbed calcium-phosphate metabolism, uremic toxins, and altered hemostasis—may further contribute to the comorbidity of CKD and CAD, possibly promoting diffuse vascular calcification and more complex atherosclerotic pathology (31, 33), yielding epicardial stenosis. Antiplatelet medications and nitrates are essential in the treatment and prevention of acute coronary syndromes (ACS) (34, 35). Antiplatelets administration may improve coronary perfusion and potentially influence perfusion imaging patterns. Nitrates decrease coronary vascular resistance, which allows resolution of microvascular obstruction and better flow (36). The mechanisms through which both medications exert effect are more suggestive of epicardial, rather than microvascular, disorder. However, the observed association between medication use and concordant SPECT–ICA results should not be interpreted as a causal effect. A plausible alternative explanation is that optimal therapy reflects a higher index of clinical suspicion and adherence to guideline-directed management.

There are several limitations inherent to our study. The observational nature of our study's design may introduce bias to the found associations. As we included only individuals who underwent both SPECT and ICA, particularly within a three-month period, individuals at a very high (>85%) or very low (<15%) CAD risk were not included as they do not require either tests for diagnosis or rule-out. However, this design enabled the study of SPECT-ICA relationship regardless of the temporal nature of CAD as a pathophysiological process. Our reliance on patients' registry which was built and operated for clinical and administrative purposes might have affected data collection. This is a real-world data study of Israeli individuals treated in a single HMO, and we were unable to account for potential minor differences in care between HMOs, though similar medical services and population is present across Israel, which suggest some consistency. Additionally, due to limitations in SPECT and ICA data acquisition processes, we could not account for important imaging factors, including difference in contrast mediums and variability among operators among others.

SPECT studies were interpreted as part of routine clinical practice, without centralized rereading or formal assessment of inter-reader variability. Information regarding the use of attenuation correction was not uniformly available and therefore could not be systematically incorporated into the analysis. Consequently, heterogeneity in imaging quality across centers—including variability in the use of attenuation correction—represents an unmeasured confounder in this study. While higher BMI was associated with increased SPECT—ICA discordance, modern SPECT systems employing attenuation correction are known to substantially reduce soft-tissue attenuation artifacts, particularly in patients with elevated BMI.

Furthermore, SPECT and ICA findings were analyzed using simplified binary definitions without incorporation of semi-quantitative SPECT parameters (e.g., Summed Stress Score or Summed Difference Score), territorial concordance between perfusion defects and coronary anatomy, or additional high-risk SPECT markers such as transient ischemic dilation, stress-induced ejection fraction changes, ischemic ECG findings, or angina during stress. Similarly, ICA positivity was defined by the presence of significant stenosis in any coronary artery without stratification by vessel, territory, or extent of disease. In addition, stenosis severity was based on visual ICA assessment without QCA or physiologic assessment, which introduces measurement error and limits inference regarding physiologic significance. In addition, both exercise-based and pharmacologic stress protocols were included without stratified analysis by stress modality, possibly contributing to inflated variability in SPECT-ICA agreement.

The number of patients in this study is small relative to the target cohort and the lack of validation by a separate group makes the observations found in this study mainly hypothesis-generating. In addition, a substantial proportion of patients with negative SPECT who underwent ICA did so as part of routine preoperative coronary evaluation prior to valvular or structural heart interventions, while only a subset were referred to ICA based on clinical judgment alone; possibly introducing verification bias.

## Conclusion

Various demographic, behavioral and clinical characteristics were associated with variations in the odds of agreement between stress SPECT and ICA among individuals who underwent both SPECT and ICA within a 3-month period. When predicting ICA results, logistic regression models that were adjusted for these characteristics had improved discrimination compared to SPECT alone, supporting the role of clinical information in the decision making regarding invasive testing.

## Data Availability

The raw data supporting the conclusions of this article will be made available by the authors, without undue reservation.
