# Supplementary material for: Clinical determinants of agreement and discordance between stress SPECT and invasive coronary angiography
Source: Front Cardiovasc Med. 2026 Feb 5;13:1701610. doi: 10.3389/fcvm.2026.1701610 (PMC12916578; doi:10.3389/fcvm.2026.1701610)
Supplement: Supplementary file 1 [file Datasheet1.pdf]

SUPPLEMENTAL APPENDIX

Supplemental Tables

Supplementary Table 1. Patient categorization according to SPECT and ICA results 2
Supplementary Table 2. Baseline demographic, clinical, laboratory, and treatment characteristics stratified by SPECT–ICA agreement groups 3
Supplementary Table 3. Predictive performance of stress SPECT and combined clinical models including HbA1c 5

Supplementary table 1: Patients' data categorization into groups

	SPECT negative	SPECT positive
ICA negative	-SPECT/-ICA (n = 50)	+SPECT/-ICA (n = 230)
ICA positive	-SPECT/+ICA (n = 61)	+SPECT/+ICA (n = 574)

Supplementary table 2: Descriptive statistics for association between single photon emission computed tomography myocardial perfusion scan and Invasive coronary angiography results

Variable	Overall (n=915)	SPECT (-), ICA (-) (n=50)	SPECT (-), ICA (+) (n=61)	SPECT (+), ICA (-) (n=230)	SPECT (+), ICA (+) (n=574)	P
Demographic characteristics						
Age (year)	915	66.62 (10.71)	66.23 (10.84)	64.23 (11.11)	65.97 (9.61)	0.13
female sex	192 (21%)	19 (38%)	13 (21.3%)	64 (27.8%)	96 (16.7%)	0.00
Physical characteristics						
BMI (kg/m^2)						0.00
14-18.49	2 (0.2%)	0 (0%)	0 (0%)	0 (0%)	2 (0.4%)	
18.5-24.9	120 (13.5%)	11 (22.4%)	14 (23%)	26 (11.9%)	69 (12.3%)	
25-29.9	369 (41.6%)	22 (44.9%)	29 (47.5%)	82 (37.4%)	236 (42.2%)	
30-45	397 (44.7%)	16 (32.7%)	18 (29.5%)	111 (50.7%)	252 (45.1%)	
Acute Coronary Syndrome	113 (12.3%)	4 (8%)	5 (8.2%)	21 (9.1%)	83 (14.5%)	0.09
Angina Pectoris	729 (79.7%)	36 (72%)	50 (82%)	187 (81.3%)	456 (79.4%)	0.49
Atrial Fibrillation	105 (11.5%)	4 (8%)	5 (8.2%)	38 (16.5%)	58 (10.1%)	0.05
Congestive Heart Failure	55 (6%)	4 (8%)	2 (3.3%)	17 (7.4%)	32 (5.6%)	0.55
COPD	140 (15.3%)	8 (16%)	11 (18%)	36 (15.7%)	85 (14.8%)	0.92
Diabetes Mellitus Type 2	436 (47.7%)	18 (36%)	34 (55.7%)	90 (39.1%)	294 (51.2%)	0.00
Cardiac Familial History	105 (11.5%)	6 (12%)	9 (14.8%)	29 (12.6%)	61 (10.6%)	0.72
Hypertension	713 (77.9%)	37 (74%)	46 (75.4%)	170 (73.9%)	460 (80.1%)	0.22
Smoking	231 (25.2%)	15 (30%)	16 (26.2%)	62 (27%)	138 (24%)	0.70
PAD	138 (15.1%)	4 (8%)	13 (21.3%)	23 (10%)	98 (17.1%)	0.02
Anemia	293 (32%)	19 (38%)	26 (42.6%)	64 (27.8%)	184 (32.1%)	0.12
Liver Dysfunction	262 (28.6%)	20 (40%)	19 (31.1%)	69 (30%)	154 (26.8%)	0.22
Renal failure	197 (21.5%)	12 (24%)	17 (27.9%)	28 (12.2%)	140 (24.4%)	0.00
Iron deficiency anemia	118 (12.9%)	12 (24.0%)	9 (14.8%)	25 (10.9%)	72 (12.5%)	0.09
Dyslipidemia	631 (69%)	38 (76.0%)	42 (68.9%)	151 (65.7%)	400 (69.7%)	0.48
Laboratory findings						
Creatinine Serum (mg/dL)	892	48 (1.58 ± 2.02)	59 (1.20 ± 0.88)	220 (1.04 ± 0.96)	565 (1.08 ± 0.57)	0.00
HbA1C (%)	729	33 (6.16 ± 1.16)	51 (6.65 ± 1.53)	171 (6.24 ± 1.00)	474 (6.73 ± 1.56)	0.00
Hemoglobin (g/dL)	894	48 (13.33 ± 1.77)	59 (13.70 ± 2.06)	221 (14.22 ± 1.60)	566 (14.06 ± 1.68)	0.00
Platelets ($\times 10^3/\mu L$)	894	48 (245.08 ± 76.46)	59 (234.59 ± 75.30)	221 (233.11 ± 66.18)	566 (232.07 ± 69.96)	0.67
Regular treatment						
NSAIDs	170 (18.6%)	7 (14%)	10 (16.4%)	42 (18.3%)	111 (19.3%)	0.77

Antiplatelets	617 (67.4%)	22 (44%)	52 (85.2%)	120 (52.2%)	423 (73.7%)	0.00
Anticoagulant	67 (7.3%)	4 (8%)	2 (3.3%)	24 (10.4%)	37 (6.4%)	0.14
Nitrates	68 (7.4%)	1 (2%)	7 (11.5%)	8 (3.5%)	52 (9.1%)	0.01
Anti-hyperlipidemic	681 (74.4%)	25 (50%)	48 (78.7%)	152 (66.1%)	456 (79.4%)	0.00
Psychiatric Medication	121 (13.2%)	7 (14%)	11 (18%)	34 (14.8%)	69 (12%)	0.48
Anti-diabetic	134 (14.6%)	4 (8%)	9 (14.8%)	24 (10.4%)	97 (16.9%)	0.06
Anti-hypertensive	720 (78.7%)	30 (60%)	52 (85.2%)	170 (73.9%)	468 (81.5%)	0.00
Diuretics	163 (17.8%)	8 (16%)	11 (18%)	42 (18.3%)	102 (17.8%)	0.99

1. SPECT, single photon emission computed tomography; ICA, invasive coronary angiography; Sig., statistical significance; PVD, peripheral vascular disease; BMI, body mass index; COPD, chronic obstructive pulmonary disease; NSIADS, non-steroidal anti-inflammatory drugs.
2. Data are mean (SD) or n (%).

Supplementary table 3: stress single photon emission computed tomography myocardial perfusion scan and models' prediction of positive Invasive coronary angiography results using clinical data including HbA1C (n=712).

Variable	SPECT only	SPECT + personal characteristics	SPECT + sig. clinical data + HBA1C (N=712)
N	915	915	712
single photon emission computed tomography	2.05 (1.37, 3.06)	1.98 (1.31, 2.99)	1.99 (1.17, 3.39)
Demographic characteristics			
Age (years)		1.02 (1.00, 1.03)	
Female sex		0.48 (0.35, 0.68)	0.47 (0.31, 0.71)
Physical characteristics			
BMI cont. (kg/m^2)			0.95 (0.91, 0.98)
Acute Coronary Syndrome			
Angina Pectoris			
Atrial Fibrillation			
Congestive Heart Failure			
COPD			
Diabetes mellitus type 2			
Familial History of cardiovascular disease			
Hypertension			
Smoking			0.70 (0.46, 1.05)
PAD			
Anemia			
Liver Dysfunction			
Renal failure – diagnosis & GFR			2.30 (1.37, 3.86)
Iron deficiency anemia combined			
Dyslipidemia Lab & diagnosis			
Laboratory findings			
Creatinine Serum (mg/dL)			
Hemoglobin (g/dL)			
HbA1c (%)			1.30 (1.12, 1.50)
Platelets ($\times 10^3/\mu L$)			
Regular treatment			

NSAIDs			
Antiplatelets			2.19 (1.50, 3.20)
Anticoagulant			
Nitrates			2.74 (1.04, 7.27)
Anti-hyperlipidemic (combined)			
Psychiatric Medication (combined)			
Anti-diabetic (combined)			
Anti-hypertensive (combined)			1.60 (1.02, 2.52)
Diuretics (combined)			0.64 (0.39, 1.06)
Sensitivity (%)	90.39	97.64	93.23
Specificity (%)	17.86	6.07	28.72
Positive predictive value (%)	71.39	70.22	77.35
Negative predictive value (%)	45.04	53.12	61.90
Accuracy (%)	68.20	69.62	75.37
AUC	0.54	0.62	0.73

1. SPECT, single photon emission computed tomography; ICA, invasive coronary angiography; Sig., statistical significance; PVD, peripheral vascular disease; BMI, body mass index; COPD, chronic obstructive pulmonary disease; NSIADS, non-steroidal anti-inflammatory drugs.
2. Associations between variables and positive ICA are presented in OR (95% CI) and are adjusted to all other variables included in the model.
